# RRM2 inhibition alters cell cycle through ATM/Rb/E2F1 pathway in atypical teratoid rhabdoid tumor

**DOI:** 10.1016/j.neo.2024.101075

**Published:** 2024-10-21

**Authors:** Le Hien Giang, Kuo-Sheng Wu, Wei-Chung Lee, Shing-Shung Chu, Anh Duy Do, Man-Hsu Huang, Yu-Ling Lin, Chia-Ling Hsieh, Shian-Ying Sung, Yun Yen, Tai-Tong Wong, Che-Chang Chang

**Affiliations:** a International Ph.D. Program for Translational Science, College of Medical Science and Technology, Taipei Medical University, Taipei 11031, Taiwan; b Department of Biology and Genetics, Hai Phong University of Medicine and Pharmacy, Hai Phong 180000, Vietnam; c Graduate Institute of Clinical Medicine, College of Medicine, Taipei Medical University, Taipei 110, Taiwan; d The Ph.D. Program for Translational Medicine, College of Medical Science and Technology, Taipei Medical University, Taipei 110, Taiwan; e Department of Physiology, Pathophysiology and Immunology, Pham Ngoc Thach University of Medicine, Ho Chi Minh City 700000, Vietnam; f Department of Pathology, Shuang-Ho Hospital, Taipei Medical University, New Taipei City 235, Taiwan; g Agricultural Biotechnology Research Center, Academia Sinica, Taipei 115, Taiwan; h General Clinical Research Center, Chung Shan Medical University Hospital, Taichung 402, Taiwan; i Institute of Medicine, Chung Shan Medical University, Taichung 402, Taiwan; j The Ph.D. Program for Cancer Biology and Drug Discovery, College of Medical Science and Technology, Taipei Medical University, Taipei 11031, Taiwan; k Pediatric Brain Tumor Program, Taipei Cancer Center, Taipei Medical University, Taipei 110, Taiwan; l Division of Pediatric Neurosurgery, Department of Neurosurgery, Taipei Medical University Hospital and Taipei Neuroscience Institute, Taipei Medical University, Taipei 110, Taiwan; m Neuroscience Research Center, Taipei Medical University Hospital, Taipei 110, Taiwan; n TMU Research Center for Cancer Translational Medicine, Taipei Medical University, Taipei 110, Taiwan; o Master Program in Clinical Genomics and Proteomics, School of Pharmacy, Taipei Medical University, Taipei 11031, Taiwan; p Ph.D. Program in Drug Discovery and Development Industry, College of Pharmacy, Taipei Medical University, Taipei 110, Taiwan; q Traditional Herbal Medicine Research Center of Taipei Medical University Hospital, Taipei 11031, Taiwan

**Keywords:** ATRT, RRM2, COH29, Cell cycle, ATM/Rb/E2F1

## Abstract

**Background:**

Atypical teratoid rhabdoid tumor (ATRT) is an aggressive brain tumor that mainly affects young children. Our recent study reported a promising therapeutic strategy to trigger DNA damage, impede homologous recombination repair, and induce apoptosis in ATRT cells by targeting ribonucleotide reductase regulatory subunit M2 (RRM2). COH29, an inhibitor of RRM2, effectively reduced tumor growth and prolonged survival in vivo. Herein, we explored the underlying mechanisms controlling these functions to improve the clinical applicability of COH29 in ATRT.

**Methods:**

Molecular profiling of ATRT patients and COH29-treated cells was analyzed to identify the specific signaling pathways, followed by validation using a knockdown system, flow cytometry, q-PCR, and western blot.

**Results:**

Elevated E2F1 and its signaling pathway were correlated with poor prognosis. RRM2 inhibition induced DNA damage and activated ATM, which reduced Rb phosphorylation to promote Rb-E2F1 interaction and hindered E2F1 functions. E2F1 activity suppression led to decreased E2F1-dependent target expressions, causing cell cycle arrest in the G1 phase, decreased S phase cells, and blocked DNA damage repair.

**Conclusion:**

Our study highlights the role of ATM/Rb/E2F1 pathway in controlling cell cycle arrest and apoptosis in response to RRM2 inhibition-induced DNA damage. This provides insight into the therapeutic benefits of COH29 and suggests targeting this pathway as a potential treatment for ATRT.

## Introduction

Atypical teratoid rhabdoid tumor (ATRT) is a highly malignant tumor of the central nervous system (CNS), mainly seen in children younger than three years old [1]. The main molecular genetic basis of ATRT involves the loss of function of *SMARCB1* or *SMARCA4* [2]. Due to the absence of established standard treatment strategies, therapeutic approaches for ATRT vary widely among institutions and countries. Improved survival outcomes have been achieved by combining surgery with intensive multimodal therapies such as chemotherapy, radiotherapy, or autologous stem cell rescue [3]. Despite medical advances, patient outcomes remain variable, with low overall survival rates [1,3].

Cell cycle dysregulation is central to cancer pathogenesis, as malignant cells cleverly circumvent apoptosis and evade cell cycle arrest to maintain their abnormal proliferation. This aberration is mainly caused by mutations or dysregulation of key proteins in signaling pathways that control the cell cycle. Therefore, cell cycle checkpoints have become critical signaling hubs in determining cell fate [4]. When DNA suffers damage or errors, cell cycle checkpoints and DNA damage response mechanisms serve as key decision points to coordinate the selection of appropriate repair pathways [5]. In the event of failure of repair mechanisms, DNA damage checkpoints induce irreversible cell cycle arrest or trigger apoptosis in response to excessive DNA damage [5,6]. This response increases the therapeutic potential of targeting cell cycle regulation, cell cycle checkpoints, and DNA damage response (DDR) mechanisms in cancer therapy [4,6], providing a promising avenue for therapeutic intervention aimed at disrupting the unbridled proliferation characteristics of cancer cells.

E2F transcription factors are known to be critical mediators of DNA replication and cell cycle progression. E2F members regulate the transcription, translation, and post-translational modification of many important cell cycle checkpoints and DNA damage checkpoint proteins [7]. Mutation or alteration of E2F-dependent targets has been found in most cancers and correlates with poor prognosis [7,8]. Sustained E2F transcription is required to maintain optimal levels of checkpoint proteins in response to replication stress, and E2F activity is the primary response mechanism to protect against DNA damage [9]. Deregulated expression of E2F target genes during S/G2 phase leads to aberrant DNA damage response, leading to cell cycle exit during G2 phase [10]. The E2F transcription factor family has eight members with complex functions: E2F1-3 acts as activators, and E2F4-8 acts as repressors. However, each E2F member exhibits distinct expression and activity throughout the cell cycle, and its functions are diverse in different cell or cancer types [7,8]. Among them, E2F1 has been described as a key regulator of DNA damage repair systems, especially for homologous recombination (HR) factors and DNA replication [11].

RRM2 is a subunit of ribonucleotide reductase (RNR) that catalyzes dNTP for DNA replication and repair [12]. The expression of RRM2 is elevated during the S phase of cell cycle and plays a vital role in DNA replication and DNA repair process [12,13]. Various studies have shown that targeting RRM2 prompts cell senescence, cell arrest, or cell death in a variety of cancers [14,15]. Furthermore, *RRM2* is considered a target gene of E2F transcription factor in certain cancers [14,16,17]. Our recent studies show that inhibition of RRM2 induces DNA damage, suppresses homologous recombination (HR) repair, and triggers apoptosis in ATRT cells [18]. In this study, we found that the E2F pathway is significantly activated in ATRT patients. Moreover, RRM2 inhibition completely blocks this pathway and induces cell cycle alteration through the ATM/Rb/E2F1 axis. These findings, together with previous evidence, elucidate the intricate interplay between DNA damage, cell cycle, and apoptosis upon RRM2 depletion in ATRT.

## Methods

### Cell culture

Human ATRT cell lines (BT12, CHLA266, and Re1P6) culture condition has been described before [18]. The Mycoplasma PCR Detection Kit (abm®) confirmed that the cells were Mycoplasma-free.

### Lentiviral transduction and cell transfection

The pLKO.1-shRNA and packaging plasmids pCMV- ΔR8.91, pMD.G were purchased from the National RNAi Core Facility (Academia Sinica, Taiwan). The lentivirus vector pLKO.1-puro carrying shRNA sequences, shE2F1 (TRCN0000332899 target sequences CAGGATGGATATGAGATGGGA), and shLuc (target sequence CTTCGAAATGTCCGTTCGGTT). Transfection, infection protocol, and shRRM2 sequences were performed as previously described [18]. Knockdown efficiency was assessed by immunoblotting using shLuc as a control.

### Cell proliferation assay

BT12 and Re1P6 knockdown cells as well as control cells were seeded into 96-well plates with 1000 and 2500 cells per well, respectively. Six replicate wells were used for each sample. The cell growth was monitored every 24 hours. To measure cell viability, 20 μL of alamarBlue reagent (Invitrogen, MA, USA, cat#DAL1100) was added to each well, and the absorbance was measured at the optical density of 570 nm using a microplate reader.

### Colony formation assay

Stable knockdown or control cells were seeded into 6-well plates with 2000 and 8000 cells per well for BT12 and Re1P6, respectively. Colonies formed after ten days were washed with PBS, fixed with 4 % formaldehyde (Macron, cat# H121-08), and stained with 0.5 % crystal violet. ImageJ software was used to count colony numbers.

### Cell cycle assay

Firstly, cells were trypsinized, washed, and counted before being fixed in 75 % ice-cold ethanol for at least 30 minutes at 4°C. After PBS washing and centrifugation, the cell pellets were re-suspended in DNA staining solution, including 100 µg/mL RNase A (BioShop, cat#RNA888) and 10 µg/mL Propidium Iodide-PI (Invitrogen, cat#P1304MP). The stained cells were incubated for 30 min at room temperature in the dark before analysis by FACS Canto II cytometer (BD Biosciences) flow cytometer. The percentage of cells in each cell cycle phase was determined using FlowJo_v10.10.0 software.

### Histological stains and Immunohistochemistry

ATRT-orthotopic brain tissue specimens were embedded in paraffin after fixing in 4 % paraformaldehyde. Tissue sections were stained with hematoxylin and eosin (HE) or used for the immunohistochemistry (IHC) analysis. Normal brain control tissue slides (HuFPT017, US Biomax, Inc., MD, USA) were used as a control in IHC staining. IHC assay was performed according to the manufacturer's instructions of Novolink Polymer Detection Systems (Leica, Germany, cat# RE7140-CE). The information for the first antibodies is listed in Supplementary Table 3. Images were captured and analyzed by Motic DSAssistance 4K (Motic, Hong Kong).

### Quantitative PCR

RNA was extracted with Trizol (Invitrogen) according to the manufacturer's protocol. cDNA was synthesized using the TOOLSQuant II Fast RT Kit (TOOLS, Taiwan). Real-time qPCR was performed using a ChamQ Universal SYBR q-PCR Master Mix (Vazyme, China) with gene-specific primer-probe sets in a LightCycler 96 (Roche, Switzerland) system. Each sample was tested with four replications. Gene-specific primer pairs were listed in Supplementary Table 1. Endogenous GAPDH was used as a standardized control for the relative changes in expression. The RT-qPCR cycling analysis was performed using a Light-Cycler 96 system.

### Western blot

The cellular extracts were treated with RIPA buffer along with Complete™ and EDTA-free Protease Inhibitor Cocktail (Roche Applied Science, Germany, cat# 4693132001). This procedure has been explained previously [18]. The primary and secondary antibodies are listed in Supplementary Table 2. The ImageJ software was used to measure the protein expression relative to the control.

### Gene expression profiles and clinical data analysis

The clinical cohorts' data, published by other studies, were analyzed using the R2 Genomics Analysis and Visualization Platform (https://hgserver1.amc.nl) and PedcBioPortal for Integrated Childhood Cancer Genomic (https://pedcbioportal.kidsfirstdrc.org). Genetic dependencies and gene expression of cell lines were analyzed using the Depmap Portal tool (https://depmap.org). Our previous published study [18,19] provides details on our ATRT patients' data and ATRT-PDX data. RNA-seq and clinical data were analyzed in an R environment. The DESeq2 tool was used for differential gene analysis, with log fold change >0 and adjusted *p*-value < 0.01. We used the differentially expressed genes (FDR<0.05) threshold as a cutoff for differential expression assessment.

### Statistical analysis

All the experiments were conducted three times to ensure accuracy. GraphPad Prism version 8.0.1 software was used to analyze the data. The results were presented as the mean with either standard deviation (SD) or standard error of the mean (SEM), depending on the case. A *p*-value of ≤ 0.05 was deemed significant. The statistical parameters and their descriptions were included in the figures and their captions.

## Results

### RRM2 inhibition alters cell cycle distribution

**Fig. 1:**
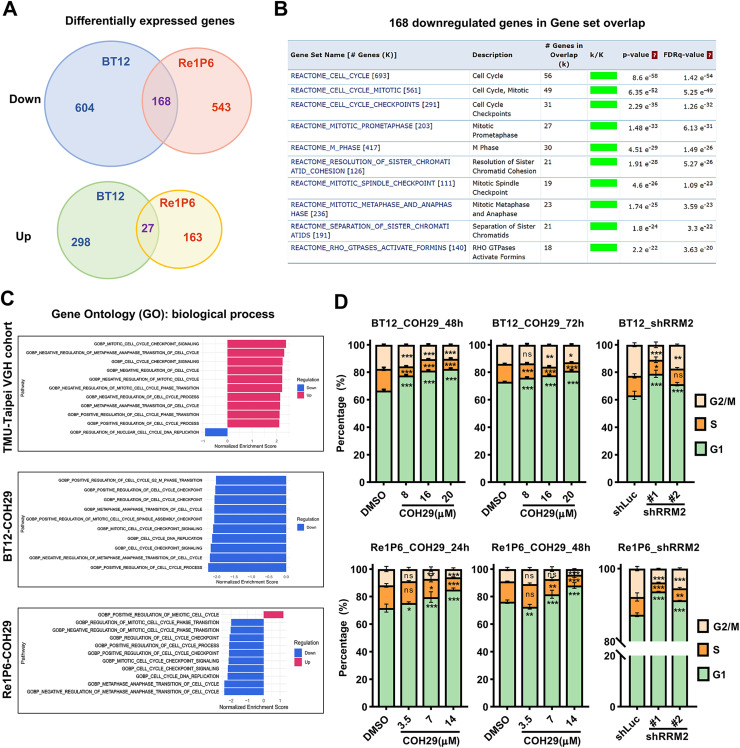
RRM2 inhibition alters ATRT cell cycle. **A** Venn diagram of significant DEGs found in BT12 and Re1P6 samples treated with COH29 versus control (DMSO 0.1 %). Genes found at the center intersection are indicated as overlap genes. Cut-off threshold: log2Foldchange >1, *p*-adjust <0.05. **B** Gene set enrichment analysis (GSEA) of the top 10 enriched pathways using 168 downregulated overlap genes of DEGs found in COH29-treated BT12 and Re1P6 cells. Data was analyzed on https://www.gsea-msig. FDR: false discovery rate. **C** GSEA of TMU-Taipei VGH cohort and two cell lines treated with COH29 in terms of normalized enrichment score (NES). Gene set collections of GO biological process were analyzed with top 10 upregulated and top 10 downregulated gene sets. **D** Statistics of the cell cycle distribution in COH29-treated cells and RRM2 knockdown cells with the controls. Cells were treated with different doses of COH29 in 24 and 48 h (Re1P6) or 48 and 72h (BT12). Data are described as the mean plus standard deviation (SD) from triplicate independent experiments. Two-way ANOVA, Dunnett's multiple comparisons test, ns represents non-significant, *p < 0.05, **p < 0.01, ***p < 0.001.

Our recent study has demonstrated that RRM2 depletion suppresses DNA damage response and induces apoptosis in ATRT [18]. However, the molecular mechanism governing the link between DNA repair defects and apoptosis remains unclear. To identify the key genes affected by RRM2 inhibition, we performed thorough RNA-seq profiling of two COH29-treated cell lines (BT12, Re1P6) compared with control DMSO-treated cells. A collection of differentially expressed genes (DEGs) found in both COH29-treated cells was collected. A total of 168 downregulated genes and 27 upregulated genes were found in both cell lines (Fig. 1A). To classify the downstream mechanism responsible for significant changes observed, we used 168 downregulated genes as input for investigative REACTOME gene set enrichment analysis (Fig. S1A). Nine of the top 10 enriched gene sets indicated cell cycle-related gene sets (Fig. 1B). This result reveals a strong effect of COH29 on ATRT cell cycle. ATRT is known as a very aggressive tumor, and the high expression of RRM2, which plays a vital role in the cell cycle, suggests that the cell cycle process may be highly activated in this tumor. To examine it, we used NGS data of the TMU-Taipei VGH cohort to analyze Gene ontology biological process (GOBP) gene set collection. The Gene Set Enrichment Analysis (GSEA) results showed that all top 10 upregulation gene sets correlate with cell cycle, including cell cycle checkpoint, cell cycle phase transition, cell cycle regulation, etc. Interestingly, those gene sets became the top 10 repressed gene sets after COH29 treatment in both BT12 and Re1P6 cells (Fig. 1C). These results were confirmed by cell cycle flow cytometry analysis. Inhibition of RRM2 by COH29 treatment or shRNA knockdown caused cell cycle arrest in G1 phase and the decreased S phase ratios (Fig. 1D, S1B). Overall, our data demonstrated that RRM2 inhibition suppressed cell cycle process and altered its distribution in ATRT.

### COH29 might alter ATRT cell cycle via E2F1 pathway

**Fig. 2:**
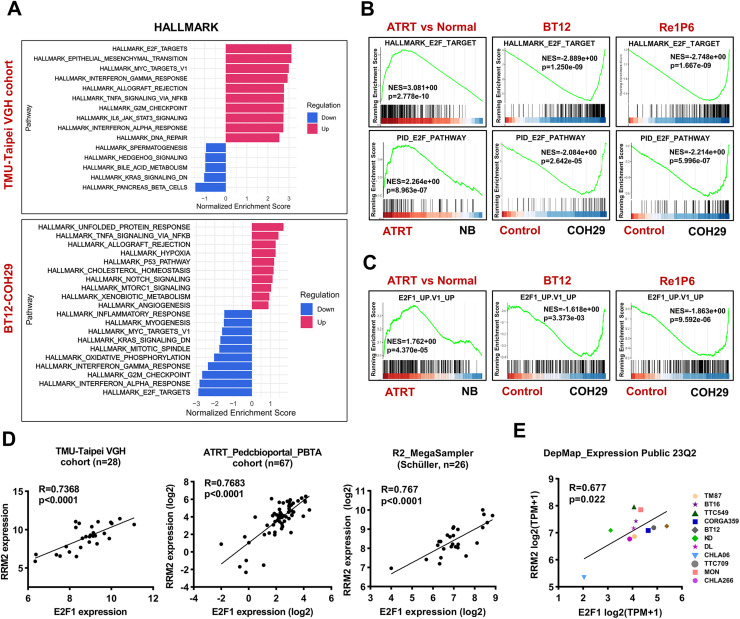
COH29 treatment suppresses E2F pathway and the positive correlation between RRM2 and E2F1 in ATRT. **A** GSEA of TMU-Taipei VGH cohort and BT12 cell line treated with COH29 in terms of normalized enrichment score (NES). HALLMARK gene set collections were analyzed with top 10 upregulated and top 10 downregulated gene sets. **B, C** Normalize enrichment score of the HALLMARK_E2F_TARGET, PID_E2F_PATHWAY **(B)** and E2F1_UP.V1_UP **(C)** gene sets in ATRT samples and COH29-treated cells (BT12, Re1P6). Normal brain (NB) or 0.1 % DMSO treatment samples were used as controls. **D** Pearson correlation test between the RNA expression level of E2F1 and RRM2 in human TMU-Taipei VGH cohort (*n* = 28), PedcBioPortal_PBTA_cohort (*n* = 67), R2_MegaSampler Schüller cohort (*n* = 26). RNA expression was normalized using logarithm base 2. **E** The correlation of mRNA gene expression between RRM2 and E2F1 in eleven ATRT cell lines. Data were analyzed from the project Expression Public 23Q2 dataset (DepMap). TPM expression values of protein-coding genes for DepMap cell lines are reported after log2 transformation and inferred from RNA-seq data using the RSEM tool with a pseudo-count of 1 (log2(TPM+1)).

To investigate the signal transduction mechanism controlling cell cycle alteration under RRM2 inhibition, we applied GSEA to the TMU-Taipei VGH cohort and COH29-treated ATRT cell lines using the HALLMARK gene sets collection. The results indicated that the TMU-Taipei VGH cohort exhibited the highest activation of the E2F-targets pathway. However, this pathway was the top-suppressed pathway under COH29 treatment in both BT12 and Re1P6 cells (Fig. 2A and S2A). The findings were further confirmed with normalized enrichment score (NES) of HALLMAK_E2F_TAGET and PID_E2F_PATHWAY gene sets (Fig. 2B). Studies have demonstrated that E2F consists of eight transcription factors from E2F1 to E2F8, which have a defined role in cell cycle control [8]. Among eight members, E2F1 has been reported as the key factor of DNA damage response through regulation HR activity [11]. To elucidate the effect of COH29 treatment on the E2F1 pathway, GSEA was applied for E2F1_UP.V1_UP gene set. The result showed that this gene set was activated in the TMU-Taipei VGH cohort (NES=1.762). However, it was repressed in COH29-treated cells (NES=-1.681 and -1.863 for BT12 and Re1P6, respectively) (Fig. 2C). Additionally, E2F1 exhibited a high correlation with RRM2 in five independent ATRT cohorts (Fig. 2D and S2B). Similar correlations were also found in 11 ATRT cell lines in the Depmap Portal database (Fig. 2E). Taken together, our data suggest that the E2F pathway is highly activated in ATRT and COH29 might suppress the E2F1 signaling pathway through the positive correlation between RRM2 and E2F1.

### High expression of E2F1 correlated with poor survival of ATRT

**Fig. 3:**
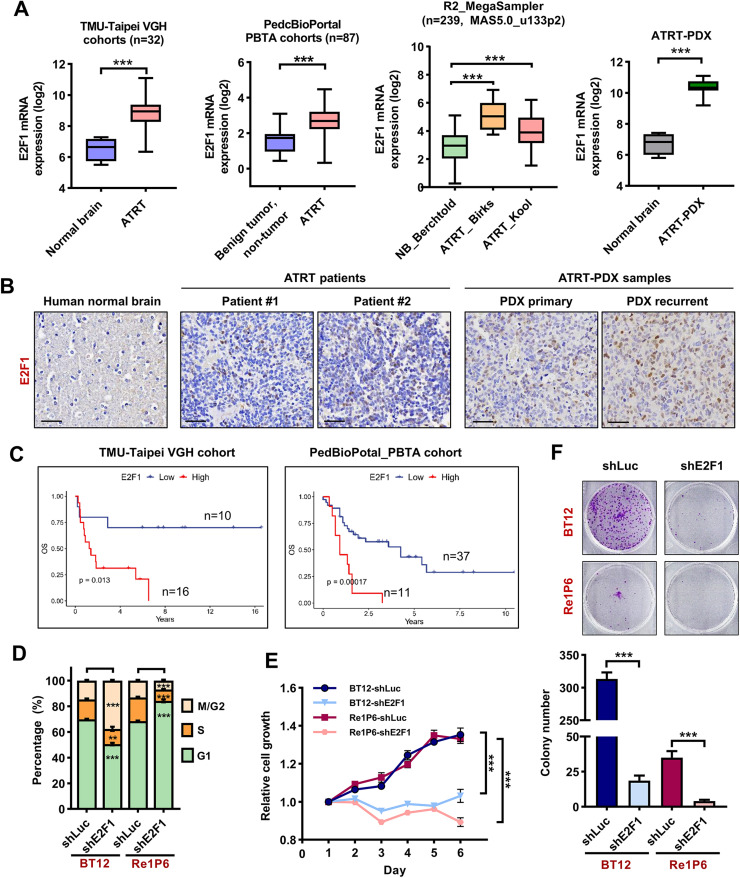
High expression of E2F1 correlated with poor survival of ATRT. **A** Expression levels of E2F1 mRNA in human ATRT and normal brain tissues/benign tumor, non-tumor in TMU-Taipei VGH cohort (*n* = 32), PedcBioPortal_PBTA_cohorts (*n* = 87), cohorts in R2 platform (*n* = 239), and ATRT-PDX sample (*n* = 23). Bar indicates the mean mRNA levels of each group; data are presented as min to max, Tukey's multiple comparisons test, ***p < 0.001. RNA expression was normalized using logarithm base 2. **B** Comparison of IHC staining for E2F1 in the normal brain, human ATRT, and ATRT-PDX samples. Scale bar 30 µm. **C** The correlation of E2F1 mRNA level with patient's overall survival (OS) in TMU-Taipei VGH cohort (*n* = 26) and PBTA cohort from PedcBioPortal (*n* = 48). **D** Statistics of the cell cycle distribution in E2F1 knockdown cells compared with the controls. **E, F** Knockdown E2F1 attenuates cell growth (**E**) and colony formation abilities (**F**) in BT12 and Re1P6 cells. Statistical significance was calculated by Two-way ANOVA, Dunnett's multiple comparisons test (cell cycle assay), and Student's t-test (cell growth and colony formation assay). All data are shown as means ± SD and performed as technical triplicates. *p < 0.05, **p < 0.01, ***p < 0.001, ns represents non-significant.

To assess the role of E2F1 in ATRT, we analyzed RNA expression of E2F1 in ATRT patient samples compared with normal brain or benign tumor/non-tumor tissues. We observed a significantly higher expression of E2F1 in five independent ATRT patient cohorts and ATRT-PDX samples (Fig. 3A). In the IHC staining, the E2F1 signal in ATRT patients and ATRT-PDX samples was stronger than in normal brain tissue (Fig. 3B). To elucidate the role of E2F1 in the growth and survival of ATRT, we firstly analyzed the correlation between the level of E2F1 and the survival of ATRT patients. All three different cohorts revealed that high levels of E2F1 strongly correlate with poor overall survival of patients (Fig. 3C and S3A).

Next, we used the shRNA knockdown system to confirm the importance of E2F1 in the growth of ATRT cells. The results of cell cycle analysis showed differences in the two cell lines (Fig. 3D and S3B). In Re1P6 cells, the knockdown of E2F1 caused G1 phase arrest and S phase depression, similar to RRM2 inhibition. In BT12 cells, we observed a decrease in both S and G1 phase populations, whereas the M/G2 phase increased. We speculate that this may be due to differences in the growth rates of the two cells. BT12 cells grow faster, so the control mechanism of E2F1 for this cell may be different from Re1P6 cells. However, S phase reduction in both E2F1 knockdown and RRM2 inhibition cells suggests the essential role of E2F1 and RRM2 in DNA replication and/or S phase entry and exit. Moreover, E2F1 depletion entirely inhibited cell proliferation (Fig. 3E) and colony formation (Fig. 3F) in ATRT cells. Remarkably, these results are similar to the cell function assay upon RRM2 inhibition [18], which again confirms the close relationship between E2F1 and RRM2 in ATRT. Together, our data indicate that E2F1 is highly expressed in ATRT and plays an important role in ATRT progression.

### COH29 treatment suppresses the expression of E2F1 and its downstream targets in ATRT

**Fig. 4:**
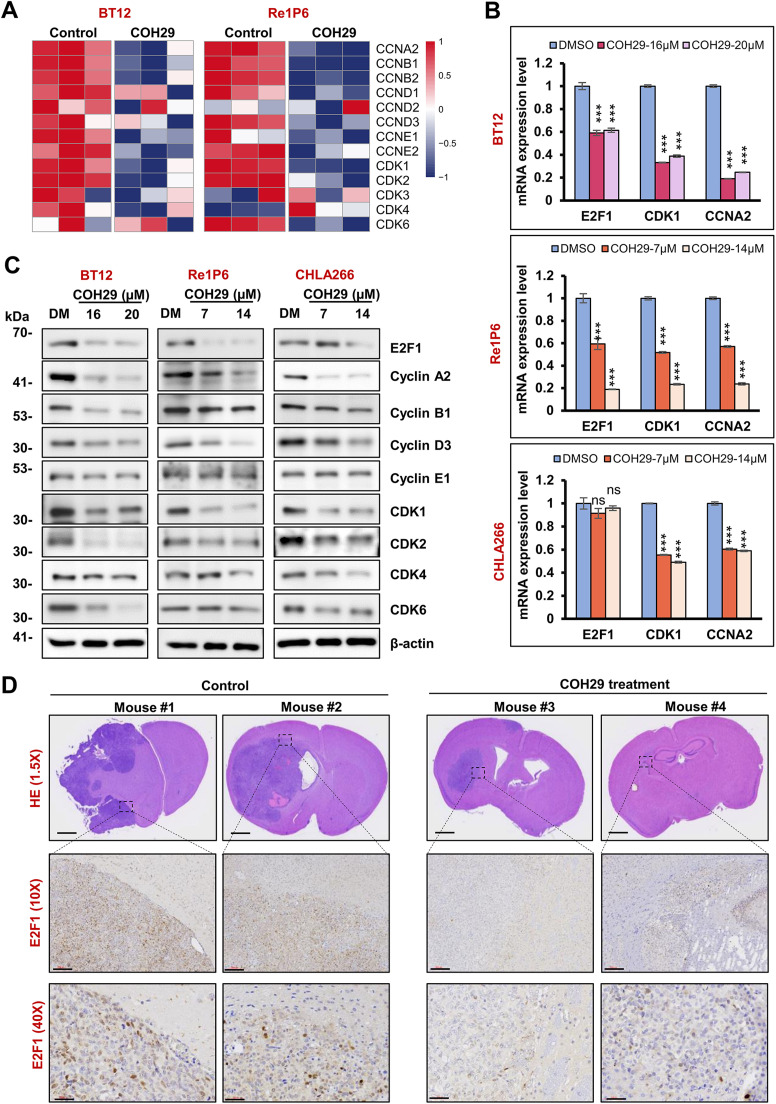
COH29 treatment suppressed the expression of cyclins and CDKs in ATRT. **A** Heatmap represents the expression of cyclins and CDKs in BT12 and Re1P6 cells treated with COH29 versus control. **B** qRT-PCR validation of E2F1, CDK1, and CCNA2 mRNA in three ATRT cell lines treated with different concentrations of COH29 compared with control. Data are described as means ± SD from triplicate independent experiments, ns represents non-significant, ***p < 0.001, Student t-test. **C** Immunoblotting for E2F1, cyclin, CDK markers in COH29-treated cells. BT12, Re1P6, and CHLA266 cells treated with DMSO (DM) or COH29 in 72 h (BT12) and 48 h (Re1p6, CHLA266), respectively. In all experiments, 0.1 % DMSO treatment was used as a control. **D** HE staining shows the size and position of the tumor in the ATRT-orthotopic mouse brain. IHC staining detects the expression of E2F1 in the ATRT xenograft brain tumor. Scale bar, from top-middle-bottom panels: 700 µm-100 µm-30 µm.

To further evaluate the impact of RRM2 inhibition on the E2F1 signaling pathway and molecular of the cell cycle progression, we analyzed mRNA sequencing data of the ATRT TMU-Taipei VGH cohort, ATRT- PDX samples, and COH29-treated cells (BT12 and Re1P6) compared with respective controls. We focused on cyclins and CDKs, which are pivotal in cell cycle control and DNA damage checkpoints. As shown in Fig. S4A and S4B, most cyclin and CDK components were upregulated in ATRT patients and ATRT-PDX samples compared to normal tissues. However, the expression levels of these genes were decreased after COH29 treatment (Fig. 4A). To validate these findings, we employed q-PCR and western blot analysis to evaluate the level of mRNAs and protein expression in three ATRT cell lines (BT12, Re1P6, CHLA266). Consistent with the reduced E2F1 expression, the level of cyclins and CDKs were reduced even with a low dose of COH29 (Fig. 4B and 4C). Our previous study demonstrated the efficacy of COH29 in inhibiting tumor growth in ATRT xenograft mice [18]. To examine the effect of COH29 on E2F1 in vivo, we used the tissue from the ATRT orthotopic model for IHC staining. The results showed that the E2F1 signal was much weaker in COH29-treated samples compared with the controls (Fig. 4D).

**Fig. 5:**
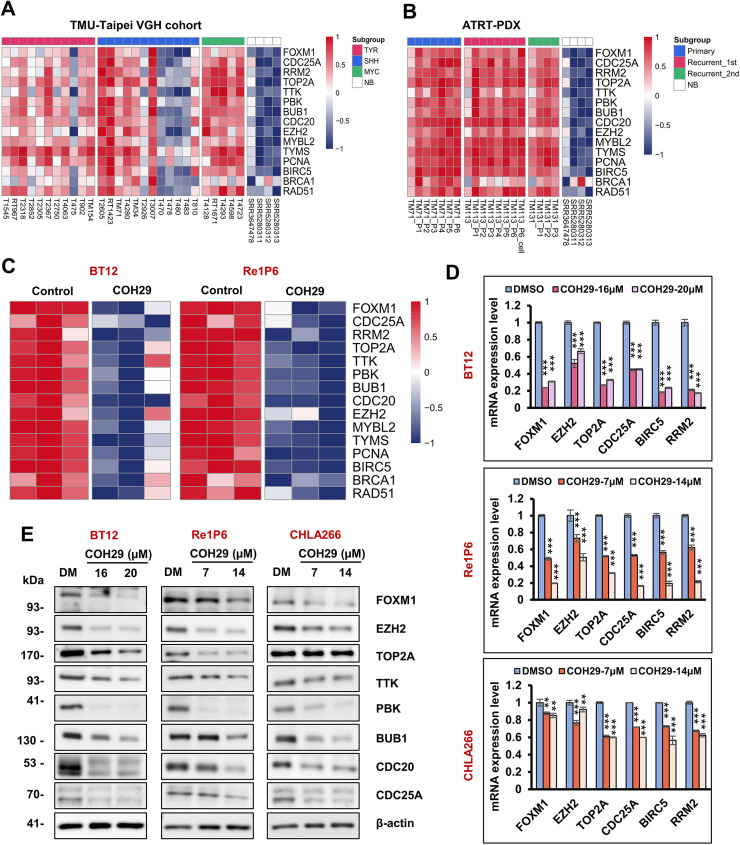
COH29 treatment suppressed the expression of E2F1-dependent target genes in ATRT. **A, B** Heatmaps represent the expression of E2F1-dependent target genes in ATRT Taipei-VGH cohort **(A)** and ATRT-PDX **(B)** samples compared with normal brain tissues. **C** Heatmap represents the expression of E2F1-dependent target genes in BT12 and Re1P6 COH29-treated cells versus control. **D** qRT-PCR validation of FOXM1, EZH2, TOP2A, CDC25A, BIRC5, and RRM2 mRNA in three ATRT cells treated with different concentrations of COH29 compared with control. Data are described as means ± SD from triplicate independent experiments, **p < 0.01, ***p < 0.001, Student t-test. **E** Immunoblotting for E2F-dependent target genes in BT12, Re1P6, and CHLA266 cells treated with DMSO (DM) or COH29 in 72 h (BT12) and 48 h (Re1P6, CHLA266). In all experiments, 0.1 % DMSO treatment was used as a control.

Given the pivotal role of E2F1 as a transcription factor controlling numerous cell cycle proteins, we analyzed the expression of downstream cell cycle-related genes. Similar to cyclins and CDKs, mRNA of 15 E2F1-target genes was highly expressed in ATRT patients and ATRT-PDX tissue compared with normal brains (Fig. 5A and 5B). Furthermore, COH29 treatment significantly suppressed the mRNA and protein expression of these genes in three ATRT cell lines (Fig. 5C–5E). Consistent with previous data, we observed a similar reduction in the expression of these proteins after E2F1 knockdown (Fig. S5A). Notably, RRM2 is a known target gene of E2F1, suggesting COH29 treatment may induce a negative feedback loop on RRM2 expression, contributing to its potent effect on ATRT even at low doses. Overall, our data indicated that COH29 alters ATRT cell cycle through suppressed E2F1 and its downstream signaling pathway.

### COH29 treatment affects the cell cycle through ATM/Rb/E2F signaling pathway in ATRT

**Fig. 6:**
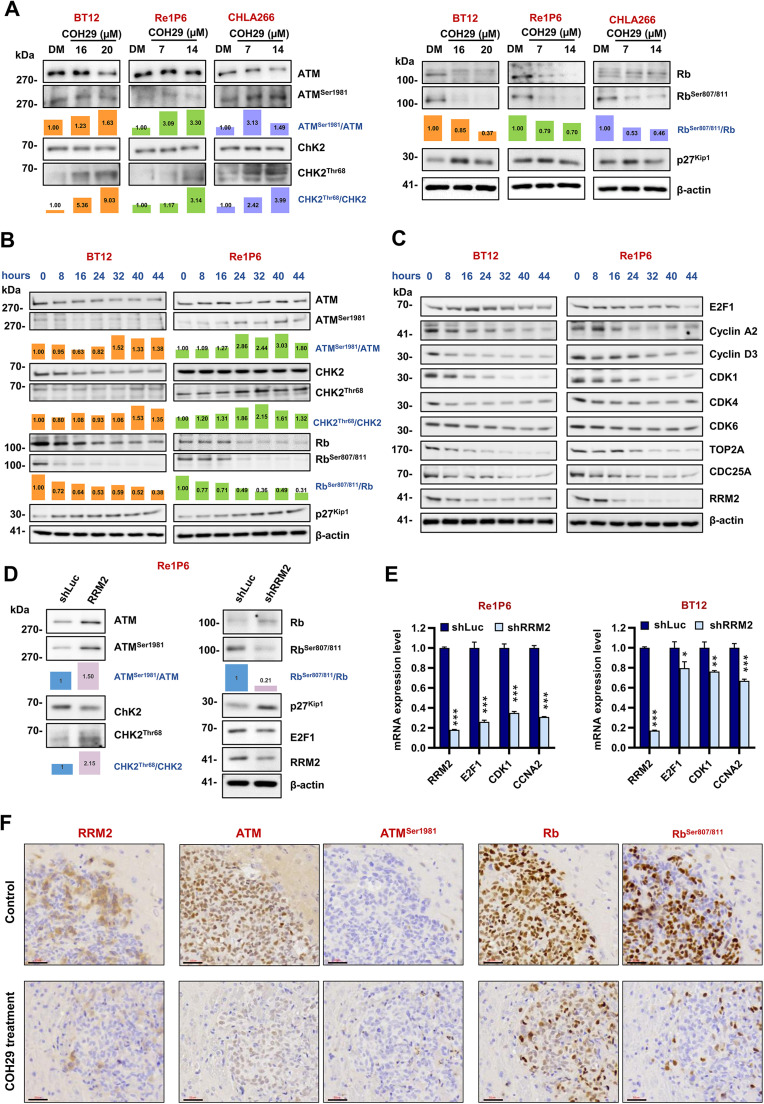
COH29 treatment alters ATRT cell cycle via ATM/Rb/E2F1 signaling pathway. **A** Immunoblotting of proteins that regulate E2F1 pathway in BT12, Re1P6, and CHLA266 cells treated with different doses of COH29: BT12 treated with 16 and 20 µM COH29 in 72 h, Re1P6 and CHLA266 treated with 7 and 14 µM COH29 in 48 h. **B, C** The time course expression level of proteins that regulate the E2F1 pathway (**B**), or E2F1-dependent target genes (**C**) in ATRT cells treated with COH29 in different time points: 0-8-16-24-32-40-44 h. BT12 and Re1P6 cells were treated with COH29 20 µM and 14 µM, respectively. **D** Immunoblotting of proteins that regulate ATM/Rb/E2F1 pathway in Re1P6 cells treated with shRRM2 or shLuc. The bar graph presents the relative expression between the protein and its phosphorylated form calculated from the displayed images. The protein expression levels were quantified using ImageJ software. The relative expression was normalized with control 0.1 % DMSO, 0 h treatment, or shLuc. **E** qRT-PCR validation of RRM2, E2F1, CDK1, and CCNA2 mRNA in Re1P6 and BT12 cells treated with shRRM2 compared with control shLuc. Data are described as means ± SD from triplicate independent experiments, *p < 0.05, **p < 0.01, ***p < 0.001, Student t-test. **F** IHC staining detects the expression of RRM2, ATM, ATM^Ser1981^, Rb, and Rb^Ser807/811^ in the ATRT xenograft brain tumor. Scale bar 30 µm.

Numerous studies have documented that the interaction between the transcription factor E2Fs and Rb is central in cell cycle control. Rb acts as a barrier to cell cycle progression by restraining E2Fs, while phosphorylation of Rb releases E2Fs to initiate the cell cycle program [20]. In light of this, we examined the protein level of Rb and pRb in three ATRT cell lines after COH29 treatment. The RRM2 inhibitor treatment dramatically reduced Rb and pRb protein levels. Notably, the ratio of pRb/Rb indicated that the decrease in pRb was sufficient to block E2F1 activity (Fig. 6A). In addition, the expression amount of E2F1 protein was reduced upon COH29 treatment (Fig. 4).

**Fig. 7:**
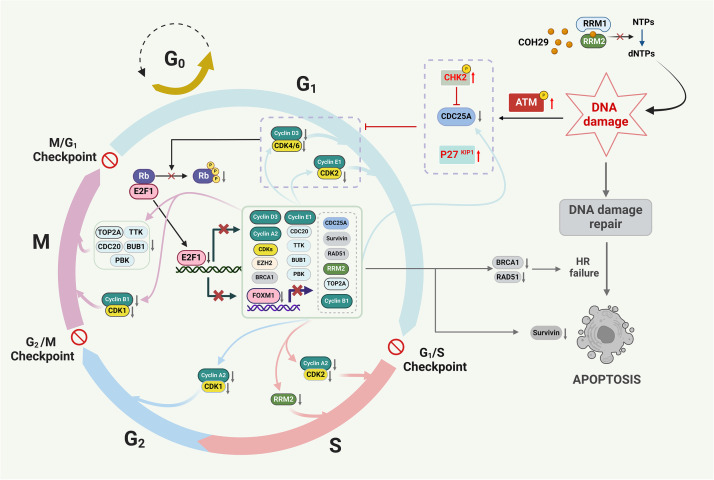
The proposed schematic illustrates the COH29 treatment induces DNA damage, alters cell cycle, and activates apoptosis via ATM/Rb/E2F1 axis in ATRT Firstly, COH29 treatment induces DNA damage in ATRT cells and activates ATM protein phosphorylation. Secondly, the phosphorylation of ATM protein regulates other proteins that are responsible for preventing Rb phosphorylation. Then, protein Rb inhibits the activity of the transcription factor E2F1. This ultimately leads to the blocking of the expression of several E2F-dependent targets. Finally, the E2F-dependent targets will alter the cell cycle, which results in the prevention of HR repair and triggers apoptosis.

In our previous investigation, we demonstrated that RRM2 depletion triggers DNA damage and suppresses the HR response in ATRT. The observed changes in cell cycle distribution and the E2F1 signaling pathway after COH29 treatment prompted us to further explore the interplay between cell cycle regulation and DNA damage response in ATRT. It is well established that ATM plays an important role in DNA damage response, and its phosphorylation can increase CHK2 phosphorylation and promote the accumulation of p27^Kip1^ [[21], [22], [23]], which is involved in regulating Rb phosphorylation [20]. Additionally, ATM activation increases CHK2 phosphorylation and reduces the cyclin E-CDK2 activity through CDC25A downregulation in response to ionizing radiation [24,25]. Cyclin-dependent kinases 4 and 6 (CDK4/6) have been shown to associate with the Rb/E2F1 complex, facilitating the phosphorylation of Rb by cyclin E-CDK2, which in turn activates E2F1 through the release of Rb [26]. Based on these findings and our previous studies, we hypothesized that RRM2 inhibition might induce DNA damage and subsequently regulate cell cycle through the ATM/Rb/E2F1 pathway. To confirm this hypothesis, we assessed the protein expression of the key components linking DNA damage and cell cycle regulation, including ATM, CHK2, and p27^Kip1^. The results showed that the phosphorylation of ATM, CHK2, and expression level of p27^Kip1^ was increased after COH29 treatment (Fig. 6A). Considering that dysregulation of cell cycle-related proteins may be due to phase transitions and not just by DNA damage, we monitored the expression of E2F1 regulatory proteins at various times after COH29 treatment. Cell lysates were collected in seven timepoints: 0-8-16-24-32-40-44 hours (Fig. 6B and 6C). In BT12 cells, a significant increase of ATM^Ser1981^/ATM and CHK2^Thr68^/CHK2 was observed after 32 hours of treatment, while p27^Kip1^ rapidly accumulated after 8 hours of treatment. Re1P6 cells were characterized by slower growth compared to BT12, with significant increases in ATM^Ser1981^/ATM and CHK2^Thr68^/CHK2 after 8h and elevated p27^Kip1^ levels observed after 24 hours. Coincidentally, both cell lines exhibited rapid depletion of Rb^Ser807/811^/Rb within 8 hours after treatment (Fig. 6B). Furthermore, E2F1 and its regulatory proteins gradually decreased over time with treatment (Fig. 6C). Despite some differences in the timing of proteins fluctuations, the final impact of COH29 on both cell lines were comparable. These results emphasize that the observed changes in protein levels indeed arise from the effects of COH29 treatment. To confirm that the decrease in Rb phosphorylation and the suppression of E2F1 were caused by RRM2 depletion rather than off-target effects of COH29, we utilized knockdown RRM2 experiments for validation. The results of RRM2 knockdown showed that the changes in gene expression were similar to those observed when cells were treated with COH29 (Fig. 6D and 6E). The findings were also validated in vivo using tissue samples from control mice and mice treated with COH29 to assess protein expression. The results indicated a marked decrease in RRM2 and Rb^Ser807/811^ signals, as well as an increase in ATM^Ser1981^ expression in the tissues from COH29-treated mice (Fig. 6F). Taken together, our study suggests that inhibition of RRM2 can induce DNA damage that subsequently affects the cell cycle through modulation of the ATM/Rb/E2F1 signaling pathway (Fig. 7).

## Discussion

ATRT is an aggressive central nervous system tumor that mostly occurs in children and has an inferior prognosis. To date, there is no specific treatment for this tumor [27]. Understanding the molecular mechanisms that control the development of ATRT may lead to new perspectives and promising therapies for this disease. In 2013, Birk et al. reported cell cycle and epigenetic dysregulation in three rhabdoid tumor subsets (two comprised of ATRT and one of kidney rhabdoid tumors) [28]. Another study using in silico analysis showed that E2F targets and cell cycle-related genes were highly enriched in three ATRT gene expression profiling datasets [29]. Consistent with these results, our data also revealed the activation of E2Fs and cell cycle signaling pathways in ATRT. In the cohort we analyzed, strongly activated E2F1 level and E2F1 signaling pathway may lead to worse patient outcomes. These data, coupled with the critical role of E2Fs in cell growth and survival, suggest that the E2F pathway may be a promising therapy for treating ATRT.

Numerous studies have reported that E2F proteins have extremely important functions in cell growth and survival [30]. Therefore, many E2F-dependent transcriptional targets have become strategic targets in cancer treatment [31]. Our recent study demonstrated that targeting RRM2, an E2F-dependent transcription target, can induce DNA damage, inhibit HR repair, and trigger apoptosis in ATRT. Besides, COH29, an inhibitor of RRM2, significantly suppressed tumor growth and prolonged survival of ATRT xenograft mice in vivo [18]. It has been reported that RRM2 is an important transcription target of E2F1 [16,17]. Comprehensibly, the activation of E2F1 may lead to the accumulation of RRM2 in ATRT. Additionally, the positive correlation between E2F1 and RRM2, along with its high expression associated with worse outcomes of patients, is reasonable. In our NGS data analysis, the E2F1_UP.V1_UP gene set was enriched in the VGH TMU-Taipei cohort, while this signaling pathway was strongly suppressed when BT12 and Re1P6 cells were treated with COH29. Captivatingly, our data showed that the RRM2 inhibition not only inactivated E2F1-dependent targets but also overwhelmed E2F1 itself. We speculate that there may be a negative feedback loop mechanism causing this situation.

E2F1 activity is believed to be tightly controlled by DNA damage checkpoints to regulate cell cycle progression and initiate cell death when necessary [32]. In this study, we explore E2F1’s role in ATRT and how its regulation affects cell behavior. Inhibition of RRM2 reduced E2F1 expression and its target genes, causing cell cycle arrest in G1 phase, decreased S phase, and apoptosis. We also evaluate the connection between DNA damage with cell cycle and apoptosis via ATM/Rb/E2F1 signaling upon COH29 treatment. ATM (protein kinase ataxia telangiectasia mutated), a key mediator of the stress response and DSB signaling, is activated by acetylation and/or phosphorylation [33]. Ser1981 phosphorylation of ATM is a marker of its activation [34]. Activated ATM phosphorylates histone H2AX at Ser139 to form γH2AX, which allows further recruitment of more DDR proteins to the damage site [35]. The phosphorylation of CHK2 at threonine 68 (CHK2^Thr68^) by ATM leads to its activation to induce G1 arrest in response to DNA damage by ionizing radiation [23,36]. CHK2 is a key component of the DNA damage response, whose substrates, such as CDC25A, CDC25C, BRCA1, and p53, have been identified to be involved in cell cycle arrest or DNA repair [37]. When exposed to ionizing radiation, degradation of CDC25A by CHK2 prevents it from activating CDK2, which plays an essential role in G1 to S transition by abrogating the S-phase checkpoint [24]. CDC25A directly or indirectly activates the cyclin E-CDK2 and cyclin A-CDK2 complexes to regulate G1–S progression [38]. Furthermore, E2F1 is also a physiological substrate of Chk2, and Chk2 is required for E2F-dependent apoptosis in response to DNA damage [39]. In our data, we observed increased phosphorylation of ATM (ATM^Ser1981^), CHK2 (CHK2^Thr68^), and decreased levels of CDC25A, CDK2, and cyclin A2 levels, which may explain why ATRT cells were arrested in G1 phase after COH29 treatment.

On the other hand, our investigation revealed an increase in p27^Kip1^ levels after COH29 treatment. Previous studies have elucidated a negative feedback loop involving cyclin A-CDK2 and p27^Kip1^, which influences E2F1 activity through the Rb phosphorylation regulation and subsequently attenuates Cyclin A transcription [40]. Moreover, CDK4/6-mediated phosphorylation of Rb plays a critical role in regulating E2F activity [20]. In our experimental results, we observed a decreased CDK4/6 protein level and reduced Rb phosphorylation. Additionally, reductions were noted in the levels of cyclin A2, cyclin D3, CDK1/2, CDK4/6, TOP2A, and CDC25A. These findings suggested that hypophosphorylated Rb leads to the formation of a complex with E2F1, thereby altering the E2F1 transcriptional activity on E2F1-dependent genes upon COH29 treatment. Furthermore, the hypophosphorylated Rb/E2F1 complex reduces the autoregulatory properties of E2F1, resulting in decreased E2F1 expression [41]. All these data contribute to explain the decreased expression of pRb, E2F1 and E2F-dependent target genes including *E2F1, CCNA2* (cyclin A2), *CCNB1* (Cyclin B1), *CCND3* (Cyclin D3), *CCNE1* (Cyclin E1), *CDK1, CDK2, CDK6, RRM2, CDC25A, CDC20, TTK, PBK, TOP2A, BUB1, EZH2, FOXM1, BRCA1, RAD51, BIRC5* (survivin), etc. These target genes have been reported to be directly or indirectly regulated by E2F1 [[42], [43], [44], [45]]. Among the E2F-dependent target genes, FOXM1, a member of the Forkhead superfamily of transcription factors, regulates target genes implicated in cancer initiation, progression, or drug resistance, such as *RRM2, BIRC5, CCNB1, PLK1, BUB1B, CENPA, KIF20A, TOP2A, BRCA2, CHEK1*, and *RAD51* [15,46]. While the functions of most of these proteins in the cell cycle and DNA damage response are well-defined, variations may occur depending on the cell type or cellular state. For instance, Cyclin D-CDK4/6 and cyclin E-CDK2 are involved in the G1/S phase transition, while cyclin A/B-CDK1/ 2 controls S phase and G2/M phase checkpoint [4]. During mitosis, CDC20, BUB1, TTK, TOP2A, and PBK play vital roles in the spindle assembly checkpoint, genome stability, and organization to ensure correct segregation for the transmission of a complete set of chromosomes to daughter cells [[47], [48], [49]]. The downregulation of these E2F1-dependent target genes upon COH29 treatment not only underscores the significant impact of RRM2 inhibition on cell cycle progression but also suggests an important role of both E2F1 and RRM2 on ATRT cell growth.

Moreover, our flow cytometry analysis results show a reduction in the proportion of S phase cells in both RRM2 inhibition and E2F1 knockdown. Our hypothesis revolves around the notion that under conditions of dNTP depletion-induced DNA damage, cells tend to accumulate in the S phase, where the majority of HR occurs. Simultaneously, RRM2 inhibition down-regulates the HR pathway, triggering cell apoptosis. The cells in the S and G2/M phases exhibit heightened sensitivity to apoptosis compared to those in the G1 phase, resulting in an elevated proportion of G1 phase cells. It is important to note that BRCA1, BRCA2, RAD51, and survivin, previously mentioned, are downstream of E2F1 and/or FOXM1, which are key components of HR and apoptosis [5,50]. This discovery aids in the interpretation of our prior findings, indicating that RRM2 inhibition induces DNA damage, blocking HR repair in ATRT cells [18]. The current trimodality therapy of ATRT in children combines tumor resection, radiotherapy, and chemotherapy. Early radiotherapy is crucial for improving survival. An effective adjusted dose of irradiation for local or metastatic disease was successfully applied in young children 1.5-3 years old and in children >3 years old. In long-term survivors, long term side effect of cranial radiation is a major concern. Given that COH29 induces DNA damage, cell cycle arrest, and apoptosis, similar to the effects of RT, the combination of COH29 with radiation-reduced therapy targeting E2F-dependent pathways may offer an effective alternative that enhances therapeutic efficacy while potentially reducing long-term side effects.

In summary, our findings highlight the significance of the ATM/Rb/E2F1 signaling pathway in coordinating DNA damage-induced cell cycle arrest and activation of apoptosis upon RRM2 inhibition. Furthermore, our study reveals a functional role of E2F1 in ATRT development. Notably, tumor cells with enhanced E2F transcription will need to activate DNA repair mechanisms to prevent excessive DNA damage. Interestingly, DNA repair pathways such as homologous recombination are transcriptionally controlled by E2Fs. Enrichment of the E2F pathway in ATRT highlights the therapeutic potential of targeting E2F-dependent regulatory genes. Understanding this molecular mechanism is important to support the clinical relevance of COH29 in ATRT therapy and warrants further investigation in clinical trials.

## Ethics approval and consent to participate

The animal experiments were conducted in accordance with ARRIVE guidelines and were approved by the Institutional Animal Care and Use Committee of Taipei Medical University (LAC-2020-0219 and LAC-2021-0517). All animal cares were performed according to the guidelines of the TMU Animal Center. This study included 28 patients was conducted in accordance with the Declaration of Helsinki, and all patients provided written informed consent. Patient information was completely anonymized. The study protocol was approved by the Institutional Review Board of Human Subjects Research Ethics Committee of the Taipei Medical University and Taipei VGH, Taiwan (IRB approval numbers: N201901033 and 2019-02-010C, respectively).

## CRediT authorship contribution statement

**Le Hien Giang:** Writing – original draft, Methodology, Investigation, Formal analysis, Data curation, Conceptualization. **Kuo-Sheng Wu:** Investigation, Formal analysis, Data curation. **Wei-Chung Lee:** Validation, Investigation. **Shing-Shung Chu:** Validation, Investigation. **Anh Duy Do:** Validation, Investigation. **Man-Hsu Huang:** Resources, Conceptualization. **Yu-Ling Lin:** Resources, Conceptualization. **Chia-Ling Hsieh:** Funding acquisition, Conceptualization. **Shian-Ying Sung:** Resources, Conceptualization. **Yun Yen:** Resources, Conceptualization. **Tai-Tong Wong:** Writing – review & editing, Supervision, Resources, Funding acquisition, Conceptualization. **Che-Chang Chang:** Writing – review & editing, Supervision, Resources, Project administration, Methodology, Funding acquisition, Conceptualization.

## Declaration of competing interest

The authors declare that they have no known competing financial interests or personal relationships that could have appeared to influence the work reported in this paper.

## Appendix Supplementary materials

Image, application 1
