# Supplementary material for: RRM2 inhibition alters cell cycle through ATM/Rb/E2F1 pathway in atypical teratoid rhabdoid tumor
Source: Neoplasia. 2024 Oct 21;58:101075. doi: 10.1016/j.neo.2024.101075 (PMC11536058; doi:10.1016/j.neo.2024.101075)
Supplement: Supplementary file 1 [file mmc1.docx]

**RRM2 inhibition alters cell cycle through ATM/Rb/E2F1 pathway in atypical teratoid rhabdoid tumor**

Le Hien Giang^1,2^, Kuo-Sheng Wu^3^, Wei-Chung Lee^3,4^, Shing-Shung Chu^3^, Anh Duy Do^1,5^, Man-Hsu Huang^6^, Yu-Ling Lin^7^, Chia-Ling Hsieh^4,8^, Shian-Ying Sung^1,4,9^, Yun Yen^10^, Tai-Tong Wong^3,11,12,13,14^*, and Che-Chang Chang^1,4,13,14,15,16,17^*

^1^ International Ph.D. Program for Translational Science, College of Medical Science and Technology, Taipei Medical University, Taipei 11031, Taiwan

^2^ Department of Biology and Genetics, Hai Phong University of Medicine and Pharmacy, Hai Phong 180000, Vietnam

^3^ Graduate Institute of Clinical Medicine, College of Medicine, Taipei Medical University, Taipei 110, Taiwan

^4^ The Ph.D. Program for Translational Medicine, College of Medical Science and Technology, Taipei Medical University, Taipei 110, Taiwan

^5^ Department of Physiology, Pathophysiology and Immunology, Pham Ngoc Thach University of Medicine, Ho Chi Minh City 700000, Vietnam

^6^ Department of Pathology, Shuang-Ho Hospital, Taipei Medical University, New Taipei City 235, Taiwan

^7^ Agricultural Biotechnology Research Center, Academia Sinica, Taipei 115, Taiwan

^8^ General Clinical Research Center, Chung Shan Medical University Hospital, Taichung 402, Taiwan

^9^ Institute of Medicine, Chung Shan Medical University, Taichung 402, Taiwan

^10^ The Ph.D. Program for Cancer Biology and Drug Discovery, College of Medical Science and Technology, Taipei Medical University, Taipei 11031, Taiwan

^11^Pediatric Brain Tumor Program, Taipei Cancer Center, Taipei Medical University, Taipei 110, Taiwan

^12^Division of Pediatric Neurosurgery, Department of Neurosurgery, Taipei Medical University Hospital and Taipei Neuroscience Institute, Taipei Medical University, Taipei 110, Taiwan

^13^Neuroscience Research Center, Taipei Medical University Hospital, Taipei 110, Taiwan

^14^TMU Research Center for Cancer Translational Medicine, Taipei Medical University, Taipei 110, Taiwan

^15^Master Program in Clinical Genomics and Proteomics, School of Pharmacy, Taipei Medical University, Taipei 11031, Taiwan

^16^Ph.D. Program in Drug Discovery and Development Industry, College of Pharmacy, Taipei Medical University, Taipei 110, Taiwan

^17^Traditional Herbal Medicine Research Center of Taipei Medical University Hospital, Taipei 11031, Taiwan.

* Corresponding authors:

To whom correspondence should be addressed. Contact should be made with:

Dr. Che-Chang Chang Ph.D, The Ph.D. Program for Translational Medicine, College of Medical Science and Technology, Taipei Medical University, 6F., Education & Research Building, Shuang-Ho Campus, No. 301, Yuantong Rd., Zhonghe Dist., New Taipei City, 23564, Taiwan.

E-mail: ccchang168@tmu.edu.tw; Tel: 886-2-66202589 ext.10602

ORCID: 0000-0001-8080-2094

Dr. Tai-Tong Wong MD., Division of Pediatric Neurosurgery, Department of Neurosurgery, Taipei Medical University Hospital and Taipei Neuroscience Institute, Taipei Medical University, No. 252, Wuxing St, Xinyi District, Taipei City, 110, Taiwan.

E-mail: ttwong99@gmail.com

**Running title:** RRM2 inhibition causes cell cycle arrest in ATRT.

**This additional file includes supplementary figures, figure legends, and tables.**

[Figure S1. RRM2 inhibition alters ATRT cell cycle.](#_Toc177894649)

[Figure S2. GSEA of HALLMARK gene set collections in Re1P6 cells and the positive correlation between RRM2 and E2F1 in ATRT.](#_Toc177894650)

[Figure S3. High expression of E2F1 correlated with poor survival of ATRT.](#_Toc177894651)

[Figure S4. Expression of cyclins and CDKs in ATRT.](#_Toc177894652)

[Figure S5. Protein expression of E2F1-dependent target genes in ATRT E2F1-knockdown cells.](#_Toc177894653)

[Supplement Table 1: List of primers information for q-PCR experiments.](#_Toc177894654)

[Supplement Table 2: List of antibodies information for Western blot experiment.](#_Toc177894655)

[Supplement Table 3: List of antibodies information for Immunohistochemistry experiment.](#_Toc177894656)

**Supplement Figure 1**

Figure S1. RRM2 inhibition alters ATRT cell cycle.

**A** Heatmap represents the expression of 168 downregulated overlap genes of DEGs found in COH29-treated BT12 and Re1P6 cells. **B** Cell cycle profiles of ATRT cells treated with COH29 or shRRM2. The indicated cells were collected and analyzed by flow cytometry.

**Supplement Figure 2**

Figure S2. GSEA of HALLMARK gene set collections in Re1P6 cells and the positive correlation between RRM2 and E2F1 in ATRT.

**A** GSEA of Re1P6 cell line treated with COH29 in terms of normalized enrichment score (NES). HALLMARK gene set collections were analyzed with the top 10 upregulated and top 10 downregulated gene sets. B Pearson correlation test between the RNA expression level of E2F1 and RRM2 in the human R2_MegaSample Kool cohort (*n* = 49) and Birk cohort (*n* = 18). RNA expression was normalized using logarithm base 2. Data were analyzed on the R2 Genomics Analysis and Visualization Platform.

**Supplement Figure 3**


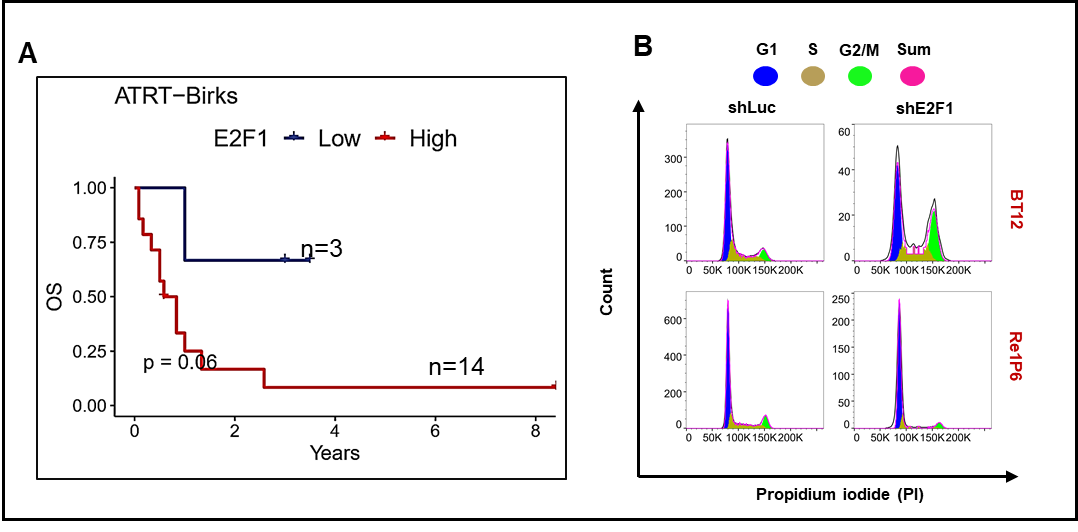


Figure S3. High expression of E2F1 correlated with poor survival of ATRT.

**A** The correlation of E2F1 mRNA level with patient’s overall survival (OS) in ATRT- Birks cohort (*n* = 17). Data were analyzed on the R2 Genomics Analysis and Visualization Platform. **B** Cell cycle profiles of ATRT cells treated with shE2F1 or shLuc control as characterized by flow cytometry.

**Supplement Figure 4**


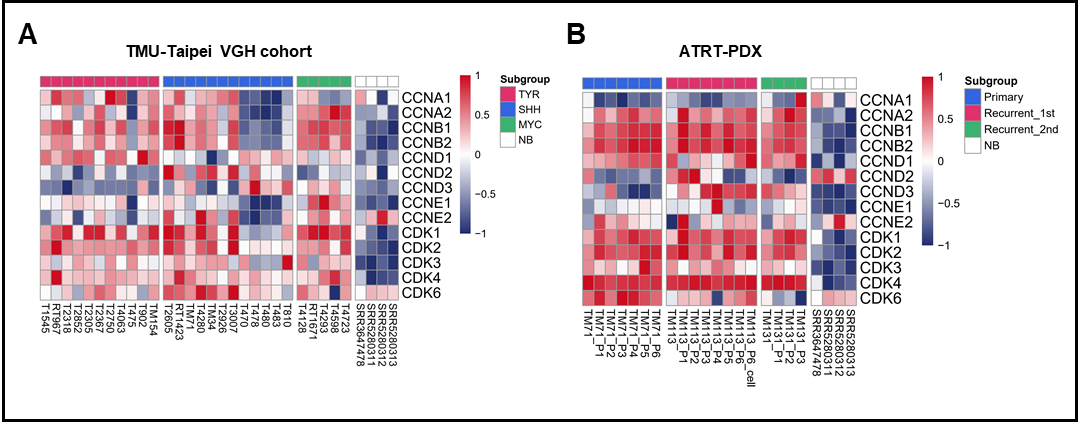


Figure S4. Expression of cyclins and CDKs in ATRT.

**A, B** Heatmap represents the expression of cyclins and CDKs in TMU-Taipei VGH cohort **(A)** and ATRT-PDX tissue **(B)** compared with normal brain (NB)

**Supplement Figure 5**

Figure S5. Protein expression of E2F1-dependent target genes in ATRT E2F1-knockdown cells.

Immunoblotting for E2F1‑dependent target genes in BT12, Re1P6 cells treated with shE2F1 or shLuc.

Supplement Table 1: List of primers information for q-PCR experiments.

| **No.** | **Primer name** | **Forward/Reverse** | **Primer sequence (from 5' to 3')** |
| --- | --- | --- | --- |
| 1 | **E2F1** | F | GGACCTGGAAACTGACCATCAG |
|  |  | R | CAGTGAGGTCTCATAGCGTGAC |
| 2 | **CDK1** | F | GGAAACCAGGAAGCCTAGCATC |
|  |  | R | GGATGATTCAGTGCCATTTTGCC |
| 3 | **CCNA2** | F | CTCTACACAGTCACGGGACAAAG |
|  |  | R | CTGTGGTGCTTTGAGGTAGGTC |
| 4 | **FOXM1** | F | TCTGCCAATGGCAAGGTCTCCT |
|  |  | R | CTGGATTCGGTCGTTTCTGCTG |
| 5 | **EZH2** | F | GACCTCTGTCTTACTTGTGGAGC |
|  |  | R | CGTCAGATGGTGCCAGCAATAG |
| 6 | **TOP2A** | F | GTGGCAAGGATTCTGCTAGTCC |
|  |  | R | ACCATTCAGGCTCAACACGCTG |
| 7 | **CDC25A** | F | TCTGGACAGCTCCTCTCGTCAT |
|  |  | R | ACTTCCAGGTGGAGACTCCTCT |
| 8 | **BIRC5** | F | CCACTGAGAACGAGCCAGACTT |
|  |  | R | GTATTACAGGCGTAAGCCACCG |
| 9 | **RRM2** | F | TTACATAAAAGATCCCAAAGAAAGG |
|  |  | R | AGCCTCTTTGTCCCCAATC |
| 10 | **GAPDH** | F | GCACCGTCAAGGGCTGAGAAC |
|  |  | R | TGGTGAAGACGCCAGTGGA |

Supplement Table 2: List of antibodies information for Western blot experiment.

| **No.** | **Name of antibody** | **Dilution** | **Catalog no.** | **Company** |
| --- | --- | --- | --- | --- |
| 1 | **Cyclin A2** | 1:5000 | GTX634420 | GeneTex |
| 2 | **Cyclin B1** | 1:2000 | 55004-1-AP | Proteintech |
| 3 | **Cyclin D3** | 1:2000 | 26755-1-AP | Proteintech |
| 4 | **Cyclin E1** | 1:1000 | A22360 | Abclonal |
| 5 | **CDK1** | 1:1000 | A0220 | Abclonal |
| 6 | **CDK2** | 1:2000 | 10122-AP | Proteintech |
| 7 | **CDK4** | 1:1000 | A11136 | Abclonal |
| 8 | **CDK6** | 1:2000 | GTX103992 | GeneTex |
| 9 | **E2F1** | 1:100 | sc-251 | Santa Cruz Biotechnology |
| 10 | **FOXM1** | 1:1000 | A2493 | Abclonal |
| 11 | **EZH2** | 1:1000 | 5246 | Cell Signaling Technology |
| 12 | **TOP2A** | 1:1000 | GTX35137 | GeneTex |
| 13 | **TTK** | 1:1000 | 10381-1-AP | Proteintech |
| 14 | **PBK** | 1:1000 | GTX60560 | GeneTex |
| 15 | **BUB1** | 1:1000 | A18053 | Abclonal |
| 16 | **CDC20** | 1:1000 | A15656 | Abclonal |
| 17 | **CDC25A** | 1:1000 | A1173 | Abclonal |
| 18 | **ATM** | 1:1000 | A19650 | Abclonal |
| 19 | **phospho-ATM-S1981** | 1:1000 | AP0008 | Abclonal |
| 20 | **CHK2** | 1:1000 | A19543 | Abclonal |
| 21 | **phospho-CHK2-T68** | 1:1000 | AP0590 | Abclonal |
| 22 | **Rb** | 1:1000 | A0003 | Abclonal |
| 23 | **phospho-Rb-S807/811** | 1:1000 | AP0484 | Abclonal |
| 24 | **RRM2** | 1:1000 | HPA056994 | Sigma-Aldrich |
| 25 | **Beta actin** | 1:10000 | 66009-1-Ig | Proteintech |
| 26 | **HRP conjugated Goat anti-Mouse** | 1:5000 | 115-035-003 | Jackson ImmunoResearch Inc. |
| 27 | **HRP conjugated Goat anti-Rabbit** | 1:5000 | 111-035-003 | Jackson ImmunoResearch Inc. |

Supplement Table 3: List of antibodies information for Immunohistochemistry experiment.

| **No.** | **Name of antibody** | **Dilution** | **Catalog no.** | **Company** |
| --- | --- | --- | --- | --- |
| 1 | **E2F1** | 1:10 | sc-251 | Santa Cruz Biotechnology |
| 2 | **RRM2** | 1:1000 | HPA056994 | Sigma-Aldrich |
| 3 | **ATM** | 1:100 | A19650 | Abclonal |
| 4 | **phospho-ATM-S1981** | 1:100 | E-AB-21540 | Elabscience |
| 5 | **Rb** | 1:1500 | 9309 | Cell Signaling Technology |
| 6 | **phospho-Rb-S807/811** | 1:500 | 8516 | Cell Signaling Technology |
